# Should patients older than 65 years be offered a second kidney transplant?

**DOI:** 10.1186/s12882-016-0426-0

**Published:** 2017-01-11

**Authors:** Kristian Heldal, Anders Hartmann, Kjersti Lønning, Torbjørn Leivestad, Anna V. Reisæter, Pål-Dag Line, Hallvard Holdaas, Karsten Midtvedt

**Affiliations:** 1 Department of Cardiology, Nephrology, Endocrinology and Geriatrics, Clinic of Internal Medicine, Telemark Hospital Trust, N-3710 Skien, Norway; 2 Department of Transplant Medicine, Oslo University Hospital, Rikshospitalet, Oslo, Norway; 3 Institute of Clinical Medicine, Faculty of Medicine, University of Oslo, Oslo, Norway

**Keywords:** Kidney transplantation, Elderly, Epidemiology, Graft survival, Chronic renal failure

## Abstract

**Background:**

Age and number of recipients in need of kidney re-transplantation are increasing. Re-transplantation practices and outcomes in elderly recipients are not previously explored. We aimed to retrospectively evaluate the outcomes of recipients 65 years and older receiving their second deceased donor allograft.

**Methods:**

The study was designed as a retrospective registry based study. All recipients 65 years or older who received a deceased donor kidney transplant at Oslo University Hospital between 2000 and 2014 were included in the study. Survival outcomes were compared between recipients of first (TX1) and second (TX2) allograft. Survival analyses were performed using the Kaplan–Meier method and Cox proportional hazard models with patient survival, uncensored graft survival and death-censored graft survival as outcomes in the analyses.

**Results:**

Seven hundred and thirty-tree recipients > 65 years received a first (*n* = 687) or second (*n* = 46) deceased donor kidney transplant. Five years uncensored graft survival rates were 64% in TX 2 and 67% in TX 1 (*P*=﻿ 0.789). Estimated five years graft survival rates censored for death with functioning graft were 88% in TX2 and 90% in TX1 (*P*=0.475). Adjusted hazard ratio for uncensored graft loss (TX2 vs. TX1) was 1.24 (95% CI 0.77 – 2.00). Adjusted hazard ratio for graft loss censored for death with functioning graft (TX2 vs. TX1) was 1.70 (0.72-4.02).

**Conclusions:**

Older recipients of second transplants have outcomes that are comparable to the outcomes of age-matched first transplant recipients, and far better than previously documented for older transplant candidates remaining on dialysis treatment. Advanced age by itself should not be a contraindication for re-transplantation. Best results are achieved with short time on dialysis before re-transplantation.

## Background

Currently the majority of patients developing end-stage renal disease (ESRD) whom are eligible for kidney transplantation are between 45 and 65 years of age [1, 2]. A kidney transplant has an expected half-life of 7–15 years [3–6]. Consequently, the majority of first time kidney transplant recipients will later in life be in need of dialysis or re-transplantation. Even though repeat kidney transplantation have lower expected graft survival compared to first kidney transplantation [7], the life expectancy in a young population is significant better than the alternative; lifelong dialysis [8]. This benefit is valid despite the fact that re-transplant recipients present a higher risk of death during the initial post-transplant phase [8].

Due to the relative scarcity of available deceased-donor kidneys, the waiting time is prolonged for most patients [9] and data from re-transplantation outcomes will provide important information for allocation policies. There are several reports supporting transplantation in older patients if they are found eligible [10–15]. An increasing number of patients will, however, be in their late 60´s and early 70´s when their first graft fails. Recipients older than 65 years who are in need of a re-transplantation constitute a unique population. They often have a history of many years with ESRD prior to their first transplantation and then long-term immunosuppressive therapy with associated comorbidities.

To the best of our knowledge there is no evidence available to support or contradict the routine of offering a second renal allograft to elderly patients with graft-failure. We believe it is important to explore this issue both for clinical and policy reasons and consequently we designed this study to evaluate the survival outcomes of kidney re-transplantation in patients older than 65 years and compare them with age- and time-matched first kidney transplant recipients.

## Methods

Data from all patients, older than 65 years at time of transplantation, who received their first or second deceased donor kidney transplant at our center between 2000 and 2014 were included in the study. Outcomes were compared between first (TX1) and second (TX2) transplants. Patient and transplant characteristics were reported prospectively to the Norwegian Renal Registry and these data were retrieved from the registry together with survival data. All patients who were included had previously given consent for the use of their clinical data in research. Last update of survival data was performed at September 1^st^ 2015.

Patients accepted for TX1 or TX2 at our center have been screened using the same age-independent algorithm. The patient´s local nephrologist performs the screening and we have no need for mandatory dialysis treatment before the patient can be accepted for the transplantation wait list. Neither age at first transplant nor graft survival of first transplant influence the decision of listing patients for second transplantation. When allocating a deceased donor kidney, we strive to achieve some degree of age matching between the donor and the recipient. As a rule of thumb, we accept a maximal age difference of 20–30 years. We do not routinely perform graft nephrectomy prior to re-transplantation.

Between 2000 and 2007, kidney transplant recipients at our center received a standard triple immunosuppressive regimen consisting of corticosteroids, Calcineurin inhibitor (CNI) with Cyclosprin A (CSA) as first choice, and either an IL2 receptor antagonist (IL2Rab) for induction therapy (2000) or mycophenolate mofetil (MMF) (2001–2007). From 2007 our standard protocol was changed to a quadruple regimen with IL2Rab induction, corticosteroids, CNI (tacrolimus or CSA) and MMF.

### Statistics

Continuous data were analysed using an independent sample Mann–Whitney test to compare the groups. A two-sided Fisher’s exact test was used in analyses of binary data. Survival analyses were performed using the Kaplan–Meier method and Cox proportional hazard models with patient survival, uncensored graft survival and death-censored graft survival as outcomes in the analyses. A *P*-value of less than 0.05 was considered as significant in all analyses. In the patient survival model, TX1 patients were censored from the analysis at time of second transplantation. Available variables with suspected association with the outcome were first tested in univariable Cox models. Variables with possible associations, defined as a *P*-value ≤ 0.2 in the univariable model, were then included in the final multivariable model. Recipient age and gender were implemented in all multivariable models regardless of the results in the univariable model. Except for three recipients with missing values for cold ischemic time (CIT), there were no missing values in the dataset for any of the variables included in the multivariable Cox models. CIT was excluded from the final model due to very low statistical significance in the preliminary univariable models. Accordingly, no patient was excluded from the final multivariable models because of missing values. All statistical analyses were performed using the statistical software package IBM SPSS 23®.

## Results

A total of 3812 kidney transplantations were performed from 2000 to 2014. Eight hundred and sixty-eight (23%) recipients were 65 years or older at time of first or second kidney transplant. Among these, 733 patients received a deceased donor kidney and were included in the final analyses. Median age was 70.9 years (range 65.0 - 82.9 years). One hundred and twenty-two patients (16.6%) received a pre-emptive transplantation, whereas 480 (65.5%) were on hemodialysis (HD) and 131 (17.9%) were on peritoneal dialysis (PD) at time of engraftment.

Forty-six patients received their second and 687 patients received their first deceased donor kidney transplant. Eight patients included in the TX2 group received their first graft between 2000 and 2014 and were at that time older than 65. They were consequently included in both groups but censored from the patient survival model at time of second transplant. Median time between first and second transplantation was 7.7 years (range 0.0 – 41.4 years). One patient was re-transplanted because of primary non-function. If this patient is excluded, median time between first and second transplantation was 8.2 years (2.5-41.4 years). Patient and transplant characteristics in TX1 and TX2 are presented in Table 1. There were some differences between the groups. We found statistical significant lower median age of 1.7 years, a lower prevalence of coronary heart disease and diabetes mellitus, higher prevalence of panel reactive antibody (PRA) positive B-cell screening and a trend towards more extensive use of older donors in the TX2 group. Underlying causes of ESRD are listed in Table 2. TX2 patients were also more likely to have ESRD caused by glomerulonephritis or polycystic kidney disease and less likely to have vascular nephropathy as cause of ESRD. The groups were comparable with respect to immunosuppressive therapy (Table 3).

**Table 1:** Patient and transplant characteristics. Continuous variables are reported as median (range)

	TX2 (*N* = 46)	TX1 (*N* = 687)	*P*-value
Age (years)	69.3 (65.1-81.6)	71.0 (65.0-82.9)	0.009
Male recipient	28 (61%)	492 (71.6%)	0.132
Pre-emptive transplantation	6 (13%)	116 (16.9%)	0.682
Coronary heart disease	4 (9%)	190 (27.7%)	0.003
Diabetes mellitus	4 (9%)	152 (22.1%)	0.039
Cerebrovascular disease	4 (9%)	81 (11.8%)	0.641
Peripheral vascular disease	5 (11%)	145 (21.1%)	0.129
Pre-emptive transplantation	6 (13%)	116 (16.9%)	0.682
Hemodialysis	34 (74%)	446 (64.9%)	0.263
Peritoneal Dialysis	6 (13%)	125 (18.2%)	0.550
Time on dialysis (months)	15.5 (0–108)	18.0 (0–87)	0.584
PRA positive B-cell screening	8 (17%)	24 (3.5%)	<0.001
CMV positive recipient	43 (94%)	568 (82.7%)	0.065
CMV positive donor	33 (72%)	513 (74.8%)	0.605
Male donor	28 (61%)	387 (56.3%)	0.645
Donor age (years)	64.1 (33.1-85.4)	61.2 (1.6-89.0)	0.106
Donor > 60 years	31 (67%)	364 (53.0%)	0.067
No HLA-A mismatch	13 (28%)	129 (18.8%)	0.124
No HLA-B mismatch	9 (20%)	77 (11.2%)	0.097
No HLA-DR mismatch	20 (44%)	318 (46.3%)	0.761
Cold ischemia time (h)	13.4 (3.8-20.2)	13.9 (2.8-29.0)	0.104

**Table 2:** Causes of ESRD

	TX 2 (*N* = 46)	TX 1 (*N* = 687)	*P*-value
Glomerulonephritis	17 (37%)	143 (20.8%)	0.016
Pyelonephritis	4 (9%)	31 (4.5%)	0.268
Polycystic kidney disease	15 (33%)	81 (11.8%)	<0.001
Vascular nephropathy	5 (11%)	264 (38.4%)	<0.001
Diabetes nephropathy	2 (4%)	62 (9.0%)	0.418
Other^a^	3 (7%)	106 (15.4%)	0.132

^a^“Other” includes interstitial, hereditary nephropathies, vasculitidis, systemic disorders, myelomatosis and 14 cases in the TX2 group with unknown cause of ESRD

**Table 3:** Immunosuppressive treatment

	TX2 (*N* = 46)	TX1 (*N* = 687)	*P*-value
Induction (IL2R)	36 (78%)	492 (71.6%)	0.398
Prednisolone	46 (100%)	687 (100%)	1.000
Tacrolimus	12 (26%)	187 (27.2%)	1.000
CsA	34 (74%)	476 (69.3%)	0.620
No CNI	0	24 (3.5%)	0.392
MMF*	45 (98%)	646 (94.0%)	0.508
mTOR inhibitor	1 (2%)	14 (2.0%)	1.000

*Thirty patients in TX1 (4.4%) and six patients in TX2 (13%) received mycophenolic acid. All other patients received mycophenolate mofetil (*p*=0.035)

Only five recipients (11%) in the TX2 group experienced a biopsy verified acute rejection episode (ARE) compared to 140 (20.4%) in the TX1 group. The difference was however not statistically significant (*P* = 0.129). Among those with an ARE, only one out of five recipients in TX2 experienced the ARE within the first 90 days compared to 86 (61.4%) in TX1. Median time from transplantation to first ARE was 163 days (21–349) in TX2 compared to 58.5 days (3–1951) in TX1, but the difference was not statistically significant (*P* = 0.190).

During follow-up there were 20 (43%) graft losses in TX2 and 327 (48%) in TX1. Death with functioning graft was the predominant cause of graft loss in both groups being responsible for 14 (70%) graft losses in TX2 and 257 (79%) in TX1. Rejection was the cause of graft loss in two patients (10%) in TX2 and 31 patients (9.5%) in TX 1. All other causes were accounted for and because of low numbers comparisons between groups are of limited value and were not performed.

**Fig. 1:**
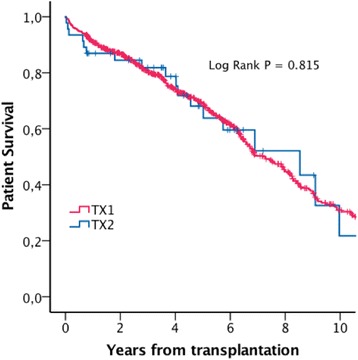
Estimated patient survival

**Fig. 2:**
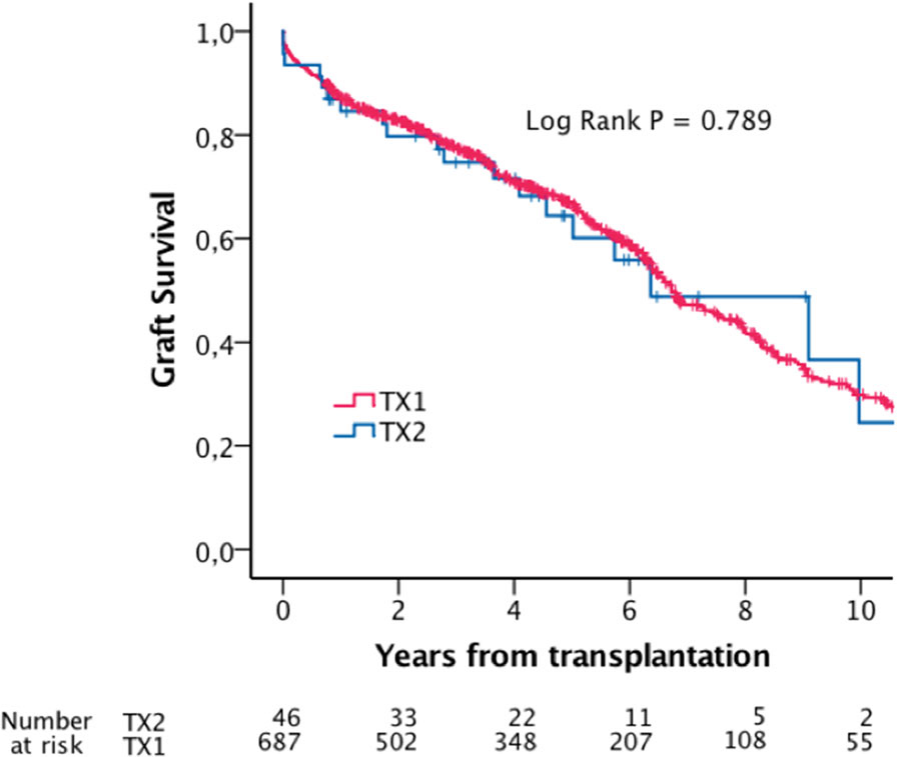
Estimated graft survival

**Fig. 3:**
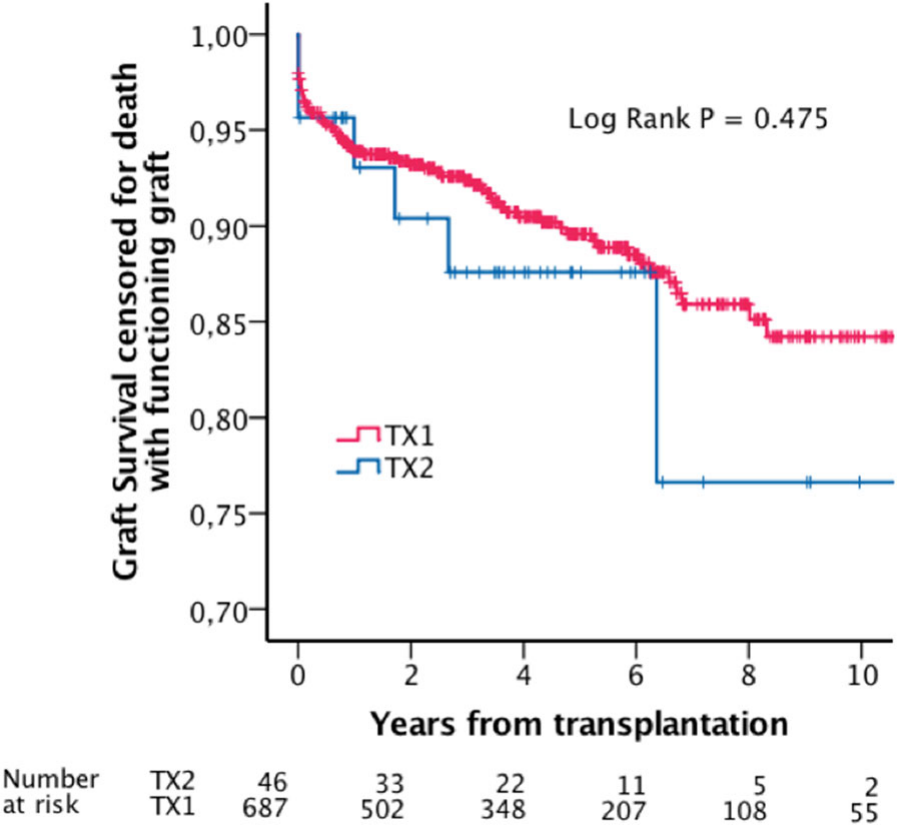
Estimated graft survival censored for death with functioning graft

Estimated mean patient survival was 7.0 (5.6-8.3) years in TX2, and 7.6 (7.1-8.1) years in TX1 (Log Rank *P* = 0.815). Estimated five-year patient survival was 68% and 69% in TX2 and TX1 respectively (Fig. 1). Estimated mean uncensored graft survival was 6.7 (5.3-8.1) years in TX 2 and 7.3 (6.8-7.7) years in TX1 (Log Rank *P* = 0.789). Estimated five year uncensored graft survival rates were 64% in TX2 and 67% in TX1 (Fig. 2). Estimated mean death censored graft survival was 9.6 (8.2-11.1) years in TX2 and 13.4 (13.0-13.9) years in TX1 (Log Rank *P* = 0.475). Estimated five years graft survival rates censored for death with functioning graft were 88% in TX2 and 90% in TX1 (Fig. 3).

**Fig. 4:**
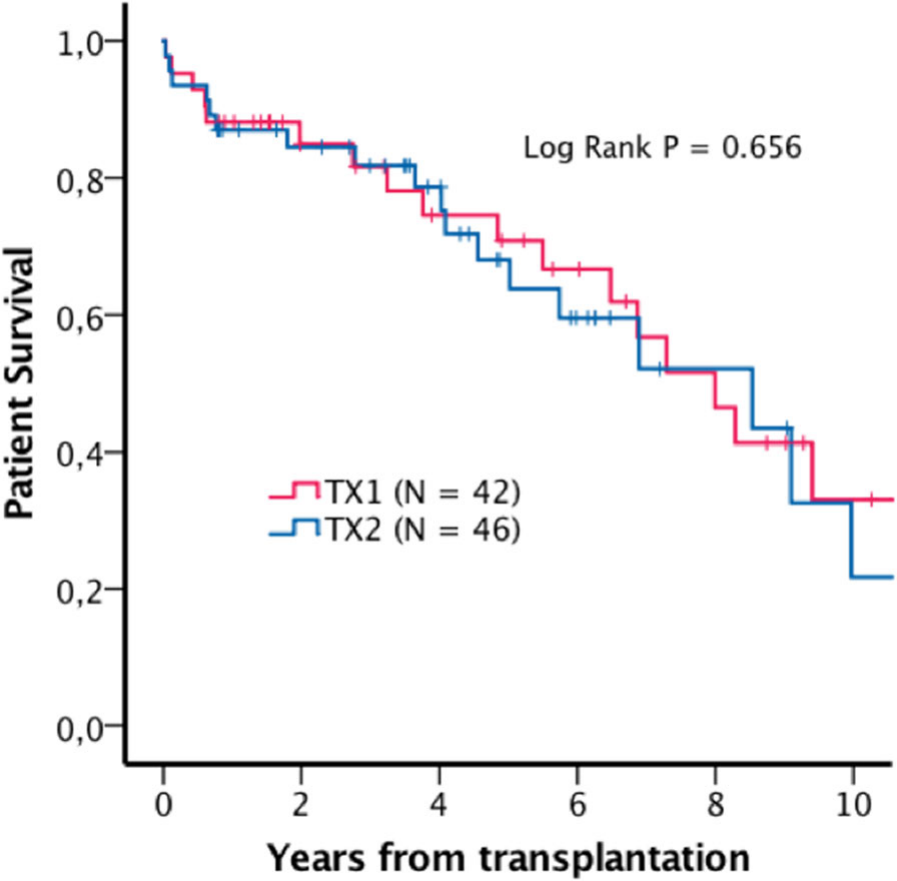
Estimated patient survival, matched dataset

**Fig. 5:**
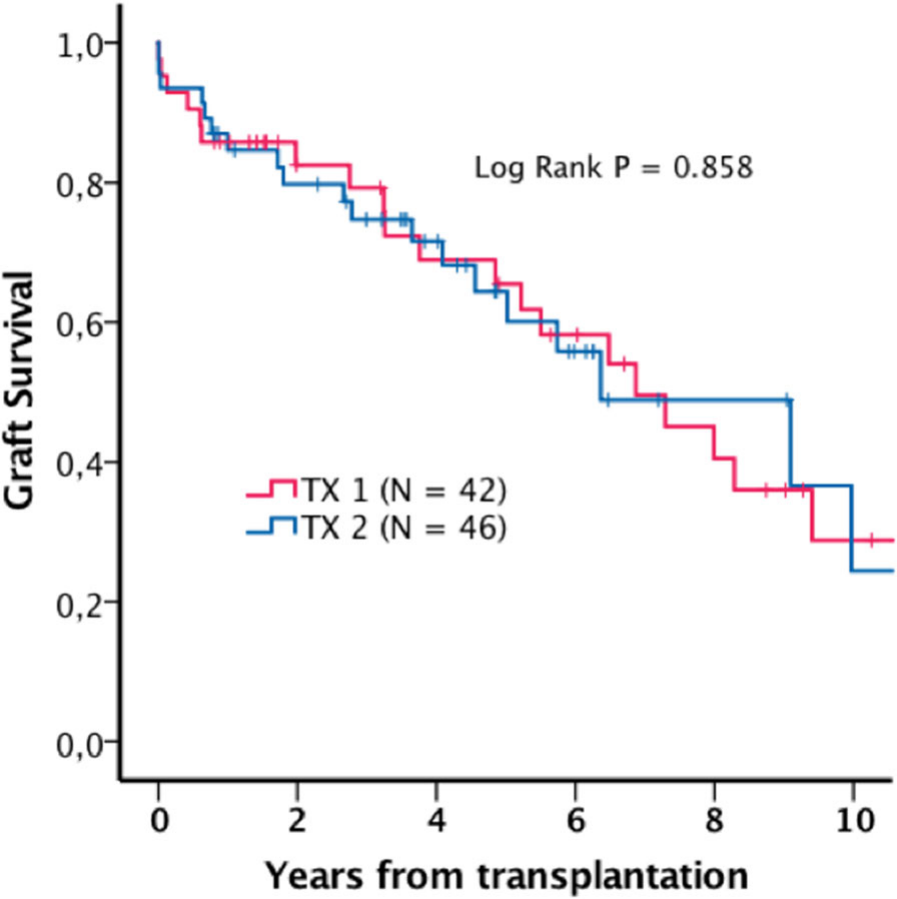
Estimated graft survival, matched dataset

**Fig. 6:**
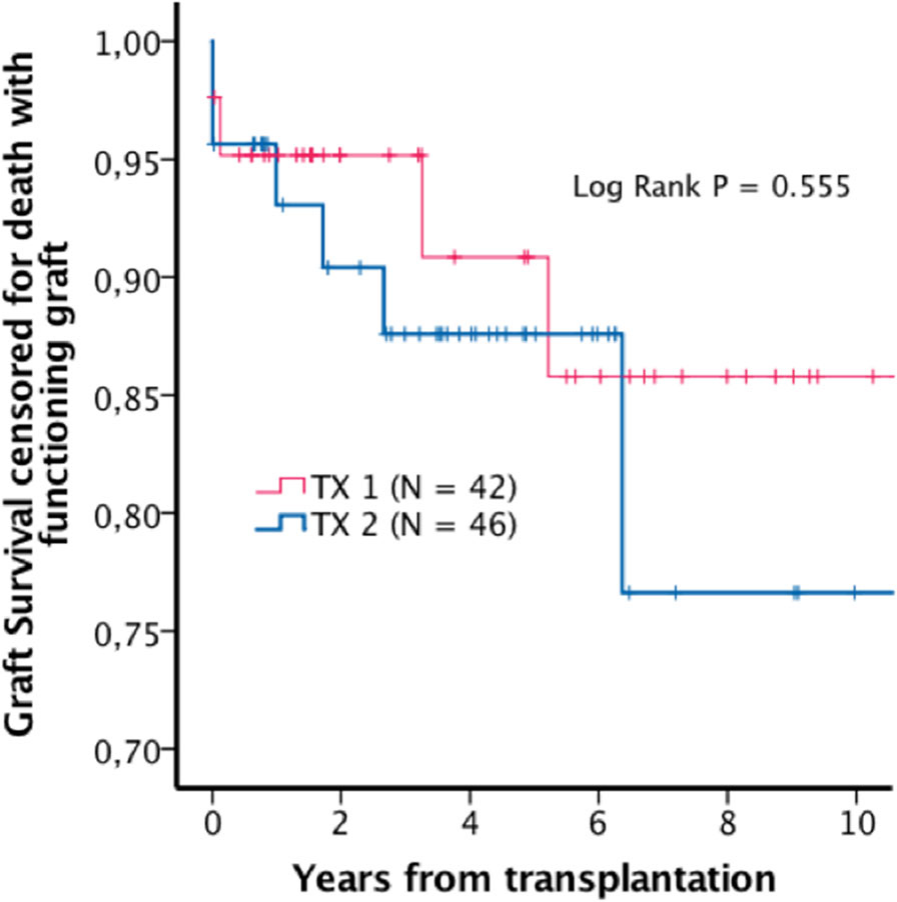
Estimated graft survival censored for death with functioning graft, matched dataset

In order to adjust for selection bias caused by differences between TX1 and TX2 in age, donor age, time on dialysis and comorbidities, a case control dataset consisting of 42 TX1 patients matched with the whole TX2 group (*N* = 46) by the following variables: age, time on dialysis, donor age > 60 years and reported prevalence of coronary heart disease, cerebrovascular disease, peripheral vascular disease and diabetes. Matching was performed using the “case control matching” syntax in the SPSS software. We managed to find only 42 case control pairs due to the matching variables we selected. The remaining four TX2 patients were however included in the analysis as they were all having more comorbidities than the rest and consequently did not contribute to any further selection bias in favor of the TX2 group. These analyses are presented in Table 4 and in Figs. 4, 5 and 6.

**Table 4:** Patient and transplant characteristics in the matched dataset. Continuous variables are reported as median (range). Survival data are reported as mean survival (95% Confidence interval)

	TX2 (*N* = 46)	TX1 (*N* = 42)	*P*-value
Age	69.3 (65.1-81.6)	69.5 (65.0-76.1)	0.808
Time on dialysis	15.5 (0–108)	13.5 (0–49)	0.345
Donor > 60 years	31 (67%)	26 (62%)	0.658
Coronary heart disease	4 (9%)	2 (5%)	0.678
Cerebrovascular disease	4 (9%)	2 (5%)	0.678
Peripheral vascular disease	5 (11%)	3 (7%)	0.716
Diabetes mellitus	4 (9%)	0	0.118
Uncensored graft survival	6.7 (5.3-8.1)	7.3 (5.6-9.1)	0.858
Death censored graft survival	9.6 (8.2-11.1)	12.0 (10.6-13.4)	0.555
Patient survival	7.0 (5.6-8.3)	8.0 (6.2-9.8)	0.656

Hazard ratio (HR) for uncensored graft loss (TX2 vs. TX1) adjusted for recipient age, recipient gender, cause of ESRD, donor age, donor source, time on dialysis, dialysis modality, prevalence of diabetes mellitus, peripheral vascular disease and HLA matching was 1.24 (95% confidence interval (CI) 0.77-2.00). The unadjusted and adjusted Cox models for uncensored graft survival are presented in Table 5. Recipient age, donor age and time on dialysis were identified as independent risk variables for uncensored graft loss whereas IL-2 induction treatment, MMF treatment and glomerulonephritis as cause of ESRD were associated with improved uncensored graft survival. Adjusted HR for graft loss censored for death with functioning graft (TX2 vs. TX1) was 1.70 (0.72-4.02). Donor age, female gender, pre-transplant diabetes and avoidance of CNI or induction treatment were identified as independent risk variables for death censored graft loss.

**Table 5:** Cox uncensored graft loss, uni- and multivariable model

	Univariable			Multivariable		
	HR	95% CI	*P*	HR	95% CI	*P*
TX2	1.06	0.68-1.67	0.789	1.24	0.77-2.00	0.375
Recipient age	1.07	1.04-1.09	<0.001	1.06	1.03-1.09	<0.001
Male recipient	1.20	0.95-1.53	0.132	1.09	0.85-1.40	0.510
Coronary heart disease	1.15	0.91-1.45	0.247			
Diabetes mellitus^a^	1.21	0.93-1.57	0.160	1.08	0.80-1.45	0.607
Cerebrovascular disease	1.27	0.92-1.75	0.143	1.14	0.81-1.59	0.462
Peripheral vascular disease	1.23	0.96-1.58	0.106	0.97	0.74-1.28	0.840
Time on dialysis (months)^b^	1.02	1.01-1.02	<0.001	1.02	1.01-1.03	<0.001
Pre-emptive transplantation^b^	0.74	0.54-1.02	0.063	0.67	0.42-1.08	0.106
HD vs. PD	1.27	0.95-1.70	0.101	1.27	0.83-1.93	0.278
Donor age^c^	1.02	1.01-1.02	<0.001	1.02	1.01-1.02	<0.001
Donor > 60 years^c^	1.54	1.24-1.91	<0.001	1.57	1.23-2.00	<0.001
Male donor	0.97	0.79-1.20	0.800			
CMV positive donor	1.23	0.97-1.58	0.094	1.06	0.81-1.39	0.664
CMV positive recipient	0.81	0.63-1.06	0.119	0.86	0.66-1.13	0.285
Cold ischemia time (CIT)	1.00	0.98-1.02	0.957			
Any HLA-A mismatch	0.78	0.60-1.00	0.05	0.73	0.56-0.96	0.025
Any HLA-B mismatch	1.28	0.91-1.80	0.160	1.42	0.98-2.07	0.066
Any HLA-DR mismatch	1.23	0.99-1.51	0.061	1.00	0.79-1.26	0.998
Induction therapy	0.82	0.66-1.03	0.089	0.67	0.52-0.87	0.003
CNI	0.88	0.55-1.42	0.605			
CSA vs. Tacrolimus	1.14	0.80-1.64	0.477			
MMF	0.77	0.55-1.08	0.123	0.61	0.42-0.90	0.011
mTOR	0.51	0.21-1.24	0.137	0.63	0.26-1.54	0.307
PRA positive	0.93	0.58-1.49	0.752			
Any acute rejection	1.11	0.87-1.42	0.393			
Glomerulonepritis	0.68	0.53-0.89	0.005	0.63	0.45-0.89	0.008
Pyelonephritis	0.90	0.56-1.47	0.687	0.65	0.37-1.13	0.124
Polycystic kidney disease	0.80	0.58-1.10	0.172	0.68	0.46-1.02	0.064
Vascular nephropathy	1.24	1.00-1.44	0.051	0.87	0.64-1.18	0.373
Diabetes nephropathy^a^	1.31	0.91-1.90	0.151	1.01	0.64-1.59	0.970

^a^Diabetes and diabetes nephropathy were tested i separate multivariable models. Diabetes was selected in the final model

^b^Pre-emptive transplantation and time on dialysis/dialysis mode were tested in separate multivariable models. In the final model, pre-emptive transplantation was excluded

^c^Donor > 60 years and Donor age were tested in separate multivariable models. Only the continuous variable Donor age was included in the final model

In a separate uncensored graft loss Cox model analyzing only TX2; age, gender, time on dialysis, donor age and induction treatment were included in the multivariable model based on the results of the univariable analyses. In this model, only recipient age (HR 1.16, 95% CI 1.03-1.31; *P* = 0.012) and time on dialysis (HR 1.03, 95% CI 1.00-1.05; *P* = 0.02) were significantly associated with inferior uncensored graft survival. For induction treatment there was a trend towards an association with improved uncensored graft survival (HR 0.32, 95%CI 0.10-1.06, *P* = 0.062).

## Discussion

In patients over 65 years we find no statistical difference in uncensored or death censored graft survival between first and second deceased donor kidney transplants. To the best of our knowledge this is the first study describing the results of re-transplantation in older recipients. With an increasing number of previous transplant recipients with failing grafts, and given the shortage of deceased donor kidneys, it is important to assess the outcome of re-transplantation in older recipients.

There was a trend towards higher donor age in the second transplant group and > 60% received a kidney from a donor older than 60 years of age i.e. an extended criteria donor (ECD). In spite of this, in the multivariable Cox models we did not find any statistically significant increased risk of graft loss in recipients of second grafts. The number of recipients in the TX 2 group was however small, and consequently the results must be interpreted with caution. This is especially true for the death censored graft loss model in which we found a statistical non-significant increased risk for graft loss of 70% in TX2 compared with TX1. This increased risk is definitely of clinical significance, and it is likely that with a larger sample size, it would reach statistical significance as well. It is therefore very likely that the lower graft survival of second grafts previously described in adult recipients [7] also is present in older recipients. On the other hand, TX2 recipients of deceased donor (DD) grafts had a five years patient survival of 68%, which is far better than expected for older patients on dialysis treatment [10, 12] and not different from five years survival of TX1 recipients. In a study evaluating 325 patients older than 60 years listed for kidney transplantation in Scotland, Oniscu et al. found a life expectancy for patients remaining in dialysis of 4.3 years after enlisting [16]. Our TX2 patients had an estimated mean survival of 6.9 years after transplantation.

Kidney re-transplantation has historically, regardless of recipient age, been associated with a poor prognosis but still provides the best chance for long-term survival and quality of life in patients facing allograft loss, as compared to maintenance dialysis therapy [8, 17–19]. Coupel et al. compared 233 second transplant recipients with 1174 first transplant recipients and observed no difference in 10-year graft survival [20]. It should be noted that the second transplant recipients were younger and had a higher level of HLA-matching than first transplant recipients. This is comparable with our findings even though we only observed non-significant differences for HLA-A and HLA-B, but no difference for HLA-DR. Arnol et al. reported a similar 15-year survival between 81 s transplant recipients compared with 427 first transplant recipients [21]. A recent large observational study with 3013 recipients including 641 s transplant recipients (the French DIVAT cohort) conclude that the risk of graft failure following a second transplantation remained consistently higher than that observed in first transplantation [18]. Contrary to our study, none of these studies specifically address increasing age at re-transplantation.

Both DGF and acute rejection episodes are known to be independent predictors of graft survival [22–24]. An interesting observation is that acute rejection episodes are less common and graft survival appears to be improved when older organs are transplanted to older recipients [25, 26]. The association between acute rejection and poor survival has also been demonstrated for recipients of a second graft [21]. In our cohort, increased immunosuppression was clearly identified as protective variables both in the uncensored (Table 4) and the death censored graft loss models. In the Cox regression model of the TX2 group, induction therapy with IL2Rab was identified as a strong (HR 0.32), but only borderline statistically significant (*P* = 0.062), protective variable. In concordance with this, our results show that with appropriate immunosuppression, graft and patient survival is good even in patients older than 65 years who receive their second transplant.

The main reason for graft loss in both groups was death with functioning graft. Graft loss due to rejection was not different between TX2 and TX1, but it must be noted that the numbers are very low in the TX2 group making it difficult to draw any firm conclusions. We were not able to detect any statistically significant differences in ARE between the groups. The study was however clearly underpowered to investigate ARE and additional studies are needed.

As always in observational studies there are several limitations. The main limitations are the small sample size, the retrospective design and the use of single centre data. Single-centre studies often end up including small numbers giving a low statistical power. Our centre has had a liberal policy regarding transplantation of the elderly [27]. Consequently we presume that few other transplant centres can provide a similar number of older second kidney graft recipients. Single centre studies also offer homogeneity with respect to medical management. This can be interpreted as a strength when comparing outcomes of first and second grafts. An additional strength is that no recipient was lost to follow-up and that the data collected from the Norwegian Renal Registry was complete and of superior quality. Further single- and multicentre studies should be performed to confirm our findings.

It is quite obvious that patients enlisted for re-transplantation at age above 65 belong to a selected population. We do however use the same age-independent screening and acceptance criteria for both first and second transplants. Consequently, those patients who are accepted for listing are accepted because they are medically fit for transplantation. In spite of this, TX2 patients had significantly lower prevalence of coronary heart disease and diabetes mellitus, indicating that TX2 patients probably were “more selected” than TX1 patients. It is difficult to select a proper group for comparison analyses. One could argue that we should have compared our second transplant recipients with age-matched recipients with failing first grafts who were on dialysis. The analyses of the matched dataset in which TX1 and TX2 patients had comparable comorbidities, age and time on dialysis did however not reveal any increased survival difference between the groups.

In a previous publication we found that 81% of dialysis patients older than 70 years who were accepted for their first kidney transplantation, eventually received a graft [10]. Consequently, the number of patients listed at our centre for a second transplant who are not receiving a graft, is very low and comparison using a Cox model with a time dependent variable (as we have used in a previous publication [10]) was evaluated as invalid. Time on dialysis is identified as a significant risk variable for survival both in this study and in previous studies [28, 29]. Due to the short waiting time at our centre, our findings may not be fully applicable to other populations of kidney transplant recipients with longer waiting times for transplantation, different clinical characteristics or different immunosuppressive regimens.

## Conclusions

Older recipients of second transplants have outcomes that are comparable to the outcomes of age-matched first transplant recipients, and far better than previously documented for older transplant candidates remaining on dialysis treatment. Best results are achieved with short time on dialysis and consequently, the transplant work-up should be initiated as soon as possible, preferably before the patient becomes in need of dialysis.

## Abbreviations

ARE: Acute rejection episode
CI: Confidence interval
CIT: Cold ischemic time
CNI: Calcineurin inhibitor
CSA: Cyclosporin A
DD: Deceased donor
DGF: Delayed graft function
ECD: Extended criteria donor
ESRD: End stage renal disease
HD: Hemodialysis
HR: Hazard ratio
IL2Rab: Interleucin 2 receptor antagonist
MMF: Mycophenolate Mofetil
PD: Peritoneal dialysis
PRA: Panel reactive antibody
TX1: First transplant recipients
TX2: Second transplant recipients

## References

[1] Matas AJ, Smith JM, Skeans MA, Thompson B, Gustafson SK, Stewart DE (2015). OPTN/SRTR 2013 Annual Data Report: kidney. Am J Transplant.

[2] Eurotransplant Annual Report 2014. 2015.

[3] Hariharan S, Johnson CP, Bresnahan BA, Taranto SE, McIntosh MJ, Stablein D (2000). Improved graft survival after renal transplantation in the United States, 1988 to 1996. N Engl J Med.

[4] Seron D, Moreso F, Arias M, Campistol JM, Curto J, Hernandez D (2011). Estimation of renal allograft half-life: fact or fiction?. Nephrol Dial Transplant.

[5] Meier-Kriesche HU, Schold JD, Kaplan B (2004). Long-term renal allograft survival: have we made significant progress or is it time to rethink our analytic and therapeutic strategies?. Am J Transplant.

[6] Sola E, Gonzalez-Molina M, Cabello M, Burgos D, Ramos J, Gutierrez C (2010). Long-term improvement of deceased donor renal allograft survival since 1996: a single transplant center study. Transplantation.

[7] Miles CD, Schaubel DE, Jia X, Ojo AO, Port FK, Rao PS (2007). Mortality experience in recipients undergoing repeat transplantation with expanded criteria donor and non-ECD deceased-donor kidneys. Am J Transplant.

[8] Rao PS, Schaubel DE, Wei G, Fenton SS (2006). Evaluating the survival benefit of kidney retransplantation. Transplantation.

[9] Schold J, Srinivas TR, Sehgal AR, Meier-Kriesche HU (2009). Half of kidney transplant candidates who are older than 60 years now placed on the waiting list will die before receiving a deceased-donor transplant. Clin J Am Soc Nephrol.

[10] Heldal K, Hartmann A, Grootendorst DC, de Jager DJ, Leivestad T, Foss A (2010). Benefit of kidney transplantation beyond 70 years of age. Nephrol Dial Transplant.

[11] Sener A, Schweitzer EJ, Munivenkatappa R, Cooper M, Bartlett ST, Philosophe B (2009). Deceased-donor renal transplantation in the geriatric population demonstrates equal graft survival compared with younger recipients. Transplantation.

[12] Rao PS, Merion RM, Ashby VB, Port FK, Wolfe RA, Kayler LK (2007). Renal transplantation in elderly patients older than 70 years of age: results from the Scientific Registry of Transplant Recipients. Transplantation.

[13] Macrae J, Friedman AL, Friedman EA, Eggers P (2005). Live and deceased donor kidney transplantation in patients aged 75 years and older in the United States. Int Urol Nephrol.

[14] Oniscu GC, Brown H, Forsythe JL (2004). How old is old for transplantation?. Am J Transplant.

[15] Wu C, Shapiro R, Tan H, Basu A, Smetanka C, Morgan C (2008). Kidney transplantation in elderly people: the influence of recipient comorbidity and living kidney donors. J Am Ger Soc.

[16] Oniscu GC, Brown H, Forsythe JL (2004). How great is the survival advantage of transplantation over dialysis in elderly patients? Nephrology, Dialysis. Transplantation.

[17] Rao PS, Schaubel DE, Jia X, Li S, Port FK, Saran R (2007). Survival on dialysis post-kidney transplant failure: results from the Scientific Registry of Transplant Recipients. Am J Kidney Dis.

[18] Trebern-Launay K, Foucher Y, Giral M, Legendre C, Kreis H, Kessler M (2012). Poor long-term outcome in second kidney transplantation: a delayed event. PLoS One.

[19] Redfield RR, Gupta M, Rodriguez E, Wood A, Abt PL, Levine MH (2015). Graft and patient survival outcomes of a third kidney transplant. Transplantation.

[20] Coupel S, Giral-Classe M, Karam G, Morcet JF, Dantal J, Cantarovich D (2003). Ten-year survival of second kidney transplants: impact of immunologic factors and renal function at 12 months. Kidney Int.

[21] Arnol M, Prather JC, Mittalhenkle A, Barry JM, Norman DJ (2008). Long-term kidney regraft survival from deceased donors: risk factors and outcomes in a single center. Transplantation.

[22] Yarlagadda SG, Coca SG, Formica RN, Poggio ED, Parikh CR (2009). Association between delayed graft function and allograft and patient survival: a systematic review and meta-analysis. Nephrol Dial Transplant.

[23] Guedes AM, Malheiro J, Fonseca I, Martins LS, Pedroso S, Almeida M (2012). Over ten-year kidney graft survival determinants. Int J Nephrol.

[24] Redfield RR, Scalea JR, Zens TJ, Muth B, Kaufman DB, Djamali A (2016). Predictors and outcomes of delayed graft function after living-donor kidney transplantation. Transpl Int.

[25] Tullius SG, Milford E (2011). Kidney allocation and the aging immune response. N Engl J Med.

[26] Chavalitdhamrong D, Gill J, Takemoto S, Madhira BR, Cho YW, Shah T (2008). Patient and graft outcomes from deceased kidney donors age 70 years and older: an analysis of the Organ Procurement Transplant Network/United Network of Organ Sharing database. Transplantation.

[27] Heldal K, Leivestad T, Hartmann A, Svendsen MV, Lien BH, Midtvedt K (2008). Kidney transplantation in the elderly--the Norwegian experience. Nephrol Dial Transplant.

[28] Heldal K, Hartmann A, Leivestad T, Svendsen MV, Foss A, Lien B (2009). Clinical outcomes in elderly kidney transplant recipients are related to acute rejection episodes rather than pretransplant comorbidity. Transplantation.

[29] Heldal K, Hartmann A, Leivestad T, Foss A, Midtvedt K (2011). Risk variables associated with the outcome of kidney recipients >70 years of age in the new millennium. Nephrol Dial Transplant.

[30] Examples of activities that do not require approval from REC. 2012. https://helseforskning.etikkom.no/ikbViewer/page/reglerogrutiner/soknadsplikt/sokerikkerek?p_dim=34999&_ikbLanguageCode=us. Accessed 21 Nov 2016.

